# Discovery of GPCR ligands for probing signal transduction pathways

**DOI:** 10.3389/fphar.2014.00255

**Published:** 2014-11-28

**Authors:** Simone Brogi, Andrea Tafi, Laurent Désaubry, Canan G. Nebigil

**Affiliations:** ^1^European Research Centre for Drug Discovery and Development (NatSynDrugs), University of SienaSiena, Italy; ^2^Department of Biotechnology, Chemistry and Pharmacy, University of SienaSiena, Italy; ^3^Therapeutic Innovation Laboratory, UMR7200, CNRS/University of StrasbourgIllkirch, France; ^4^Receptor Signaling and Therapeutic Innovations, GPCR and Cardiovascular and Metabolic Regulations, Biotechnology and Cell Signaling Laboratory, UMR 7242, CNRS/University of Strasbourg – LabEx MedalisIllkirch, France

**Keywords:** G protein-coupled receptors, GPCR, homology modeling, high throughput docking, biased agonists, biased signaling, allosteric modulators, bivalent ligands

## Abstract

G protein-coupled receptors (GPCRs) are seven integral transmembrane proteins that are the primary targets of almost 30% of approved drugs and continue to represent a major focus of pharmaceutical research. All of GPCR targeted medicines were discovered by classical medicinal chemistry approaches. After the first GPCR crystal structures were determined, the docking screens using these structures lead to discovery of more novel and potent ligands. There are over 360 pharmaceutically relevant GPCRs in the human genome and to date about only 30 of structures have been determined. For these reasons, computational techniques such as homology modeling and molecular dynamics simulations have proven their usefulness to explore the structure and function of GPCRs. Furthermore, structure-based drug design and *in silico* screening (High Throughput Docking) are still the most common computational procedures in GPCRs drug discovery. Moreover, ligand-based methods such as three-dimensional quantitative structure–selectivity relationships, are the ideal molecular modeling approaches to rationalize the activity of tested GPCR ligands and identify novel GPCR ligands. In this review, we discuss the most recent advances for the computational approaches to effectively guide selectivity and affinity of ligands. We also describe novel approaches in medicinal chemistry, such as the development of biased agonists, allosteric modulators, and bivalent ligands for class A GPCRs. Furthermore, we highlight some knockout mice models in discovering biased signaling selectivity.

## INTRODUCTION

G protein-coupled receptors (GPCRs) use canonical (G protein-mediated) and non-canonical (G protein-independent, β-arrestin dependent) signaling pathways to assert their biological functions (Luttrell et al., 1999; Beaulieu et al., 2005; Lefkowitz and Shenoy, 2005; Abbas and Roth, 2008).

The ligands can bind to receptor either competitively (orthosterically) by interacting with the same receptor-binding site as the endogenous agonist or allosterically by exerting effects through a distinct binding site. Ligands binding at the orthosteric sites have been classified as agonists, antagonists, and/or inverse agonists based on their ability to mainly modulate G protein signaling. The ligands can directly stabilize the “active” receptor conformations via a non-standard binding site (known as allosteric agonism) or modulate the binding of orthosteric ligands (known as allosteric modulation). Those ligands acting outside the orthosteric hormone binding sites can selectively engage subsets of signaling responses as “functional selectivity” or “ligand-biased signaling” (Khoury et al., 2014).

Several studies have shown that multivalent ligands, but not a monovalent ligands bind to the extracellular domains of receptors and trigger intracellular signaling by ligand-promoted receptor clustering (Sigalov, 2012). Ligands can be monovalent or bivalent, targeting specific GPCR dimers that may provide drugs with enhanced potency, selectivity, and therapeutic index. Biased ligands at GPCRs preferentially stimulate one intracellular signaling pathway over another (Violin et al., 2014). This functional selectivity of the ligands is extremely useful for elucidating the signal transduction pathways for both the therapeutic actions and the side effects of drugs. There is growing interest in developing biased GPCR ligands to yield safer, better tolerated, and more effective drugs.

Here, we discuss the discovery of GPCR ligands including biased agonists, allosteric modulators, and bivalent ligands and biased signaling selectivity for the class A GPCRs focusing on structure-based drug design (SBDD) and *in silico* screening (High Throughput Docking), medicinal chemistry, and genetic loss-of-function strategies.

## STRUCTURE-BASED DRUG DESIGN AND *IN SILICO* SCREENING (HIGH THROUGHPUT DOCKING) IN GPCRs DRUG DISCOVERY

**FIGURE 1:**
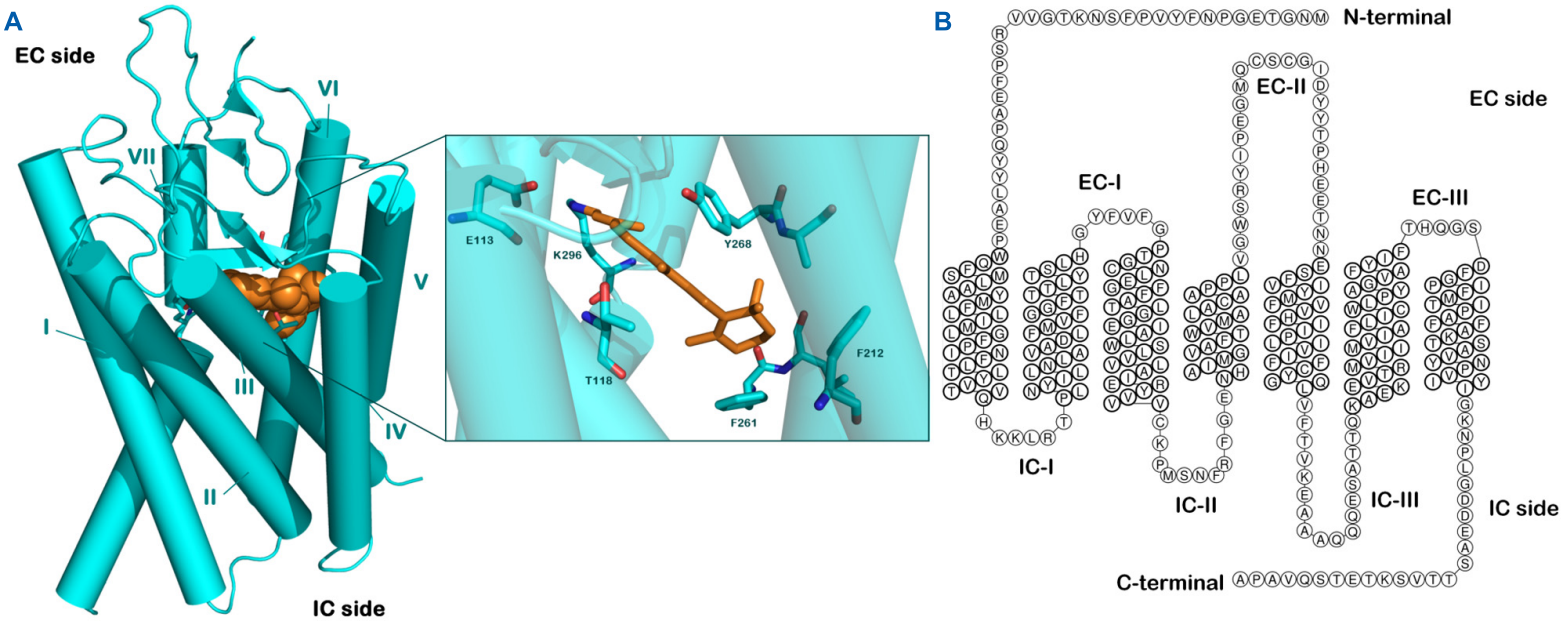
**Structure of rhodopsin. (A)** Crystal structure of bovine rhodopsin covalently linked with retinal adapted from PDB file 1F88. **(B)** Snake-like diagram for the bovine rhodopsin highlighting extracellular (EC) and intracellular (IC) loops.

Computational methods represent invaluable tools in medicinal chemistry, including drug discovery step. Concerning the ligand discovery in GPCRs field, different techniques have been applied for selecting potential and selective chemical derivatives that bind to GPCRs (Andrews et al., 2014). Homology modeling and ligand screening, utilizing structure-, and/or ligand-based approaches represent the most common approaches to discover *in silico* novel ligands. Recently, fragment-based protocols have also been used. The impact of computational techniques in GPCR drug discovery has been relevant, due to the extreme difficulties for obtaining experimental high-resolution structural information on the active and inactive state of GPCRs. After the crystallization of the first mammalian GPCR (bovine rhodopsin; Palczewski et al., 2000; **Figure 1**), homology-modeling technique has been extensively adopted to predict structures and functions of different GPCRs and also to perform *in silico* screening.

In fact, sequence analysis suggested that family A GPCRs share the same arrangement, showing a high sequence similarity of the seven transmembrane helices, confirming the suitability of rhodopsin as a template (Li et al., 2010). During the last decade, we have seen a dramatic improvement in crystallization methods. Indeed, after about 7 years from the first solved structure of a mammalian GPCR, several three-dimensional structures have been published. The second crystallized GPCR was β_2_-adrenergic receptor (β_2_AR; Cherezov et al., 2007; Rasmussen et al., 2007) and then the β_1_AR (Warne et al., 2008). Subsequently, an exponential growth of crystallized GPCR structures in the protein data bank was observed. Actually, the three dimensional structures available of class A GPCRs comprise: the adenosine A_2A_ receptor (Jaakola et al., 2008), the D_3_ dopamine receptor (Chien et al., 2010), the chemokine receptors CXCR1 (Park et al., 2012), CXCR4 (Wu et al., 2010), and CCR5 (Tan et al., 2013) the histamine H_1_ receptor (Shimamura et al., 2011), the sphingosine 1 phosphate receptor (Hanson et al., 2012), the M_2_ and M_3_ muscarinic receptors (Haga et al., 2012; Kruse et al., 2012), the μ, k, and δ opioid receptors (Manglik et al., 2012; Wu et al., 2012; Fenalti et al., 2014) as well as the nociceptin receptor (NOP; Thompson et al., 2012), bovine opsin receptors (Park et al., 2008; Scheerer et al., 2008), neurotensin receptor (White et al., 2012), the serotonin 5HT_1B_ and 5HT_2B_ receptors (Wacker et al., 2013; Wang et al., 2013a), protease-activated receptor 1 (PAR1; Zhang et al., 2012), the smoothened receptor (SMO; Wang et al., 2013b), and P2Y12 receptor (Zhang et al., 2014). Very recently, also a crystal structure of class B and C GPCRs such as glucagon receptor (Siu et al., 2013), corticotropin-releasing factor 1 (CRF_1_) receptor (Hollenstein et al., 2013) and metabotropic glutamate receptor 5 (Dore et al., 2014) respectively, have been reported.

These achievements are largely attributable to the application of high-throughput methods for lipidic cubic phase (LCP) crystallography (Cherezov et al., 2004) and protein engineering with the generation of GPCR-T4 lysozyme (Rosenbaum et al., 2007) and GPCR–BRIL fusion proteins (Chun et al., 2012). Thermo stabilization (Serrano-Vega et al., 2008) methods represent another useful tool appropriate to GPCRs crystallization. Notably, these techniques can be generally applicable to structurally diverse GPCRs. Moreover, a relevant number of receptors have been solved with both bound antagonists and agonists.

The availability of numerous different GPCR templates offers diverse options in GPCR modeling. In particular, the application of multiple templates to the homology modeling protocols has been demonstrated to improve the reliability of the computational models including GPCRs (Fernandez-Fuentes et al., 2007; Mobarec et al., 2009; Sokkar et al., 2011; Cappelli et al., 2013; Gemma et al., 2014b).

In conclusion, the availability of a relevant number of crystal structures improves results of homology modeling procedures by using novel methodology such as multiple-templates based alignment for building the structure of GPCRs as well as the three-dimensional structure of any type of proteins (Cappelli et al., 2013; Gemma et al., 2014a; Giovani et al., 2014). Moreover, the accessibility of GPCR crystal structures unlocked opportunities to use alternative methods for GPCR drug discovery, mainly SBDD. SBDD approaches are extensively used in drug discovery of novel compounds based on three dimensional protein structures using various computational methods. The impact of GPCR crystal structures on SBDD has been instantaneous and has led to the discovery of novel ligands for different GPCRs (Kooistra et al., 2013). Furthermore, as above mentioned the increase of GPCR determined structures assures a large number of potential available templates, guaranteeing an improvement of quality of GPCR homology models for virtual screening. Indeed, virtual screening has become a routine tool for selecting putative lead compounds and identifying potential drug candidates for a given target (Brogi et al., 2009, 2011, 2013; Castelli et al., 2012). Although ligand-based methods were found to be useful for structurally non-characterized targets, high throughput docking is clearly the most popular approach used in receptor based virtual screening using both experimental and theoretical sources (Abagyan and Totrov, 2001). In this review, we will present an overview of the most relevant structure-based approaches for identifying novel ligands, targeting allosteric, and/or orthosteric binding sites, for some of the class A GPCRs.

### DRUG DESIGN AND DISCOVERY IN CARDIOVASCULAR DISEASES

#### *β*-adrenergic receptors

The selectivity of compounds for β_1_- and β_2_-(AR) is an important issue to take into account in current adrenoreceptor drug design. A structure-based design approach using protein–ligand crystal structures of the β_1_AR is the first example of GPCR crystallography with ligands derived from fragment screening. In fact, the structures of the stabilized β_1_AR in complex with two ligands were determined at resolutions of 2.8 and 2.7 Å, respectively (Christopher et al., 2013). A very elegant work has been recently carried out by Christopher et al. (2013) using biophysical fragment screening of a thermostabilized β_1_AR. They also applied surface plasmon resonance (SPR) to identify moderate affinity, high ligand efficiency (LE) arylpiperazine hits. Subsequent hit to lead follow-up confirmed the activity of the chemotype. Vilar et al. (2010) evaluated the applicability of ligand-based and structure-based models to quantitative affinity predictions and virtual screening for ligands of the β_2_AR.

The crystal structure of β_2_AR has been used by Kolb et al. (2009) to investigate the advantages and limitations of the structure-based approach in ligand discovery. The authors docked about 1,000,000 commercially available compounds against the β_2_AR structure. Twenty five hits have been selected and submitted to biological evaluation. Six compounds were active with binding affinities <4 μM, with the best molecule that showed a Ki of 9 nM. Moreover, five of these molecules have been found as inverse agonists (Kolb et al., 2009).

Sabio et al. (2008) and Topiol and Sabio (2008) performed a high-throughput docking with proprietary and commercial databases to investigate the usefulness of crystal structure for discovery of novel chemical classes acting as β_2_AR inhibitors. These findings were further validated using X-ray structures of β_2_AR/Timolol (Hanson et al., 2008), via *in silico* high-throughput docking of proprietary and commercial databases. This study resulted in the identification of ligands with relevant affinity for β_2_AR (Sabio et al., 2008; Topiol and Sabio, 2008).

More recently, Weiss et al. (2013) reported a prospective, large library virtual screen of 3.4 million molecules, yielding four full agonists and two partial agonists. The exploration of features that confer selectivity to the designed compounds has also been investigated by Xing et al. (2009). The authors developed a selective pharmacophore model based on a series of selective β_2_AR agonists, presenting the first study using a ligand-based computational approach to generate specific three-dimensional pharmacophore hypotheses for the β_2_AR from its selective agonists. The best pharmacophore hypothesis consisted of five chemical features (one hydrogen-bond acceptor, one hydrogen-bond donor, two ring aromatic, and one positive ionizable feature). The result was in accordance with the reported interactions between the β_2_AR and agonists. Interestingly, the pharmacophore hypothesis can perfectly differentiate β_2_-agonists from β_1_-agonists, providing a valuable tool for virtual screening to find selective compounds against β_2_AR.

#### Endothelin receptors

In mammals, endothelins (ETs) are potent regulators of vessel functions involved in the pathophysiology of cancer, congestive heart failure, cardiovascular, proteinuria, and glomerulosclerosis. These peptides (ET_1-3_) exert their biological effects via activation of four ET receptors, ET_A_, ET_B1_, ET_B2_, and ET_C_. Activation of the ET_A_ receptor is associated with pronounced vasoconstriction whereas ET_B_ receptor occupation is linked to vasodilation. In addition, other subtypes of the ET_B_ receptor exist, one mediating vasodilation (ET_B1_) and the other eliciting constriction (ET_B2_). An additional receptor subtype, ET_C_, has been identified although its physiological significance is uncertain (Pollock et al., 1995)

Funk et al. (2004) applied a pharmacophore model of endothelin-A (ET_A_) selective receptor antagonists for screening a chemical database and identified two structurally novel lead compounds with satisfactory affinity for ET_A_ receptor.

#### Angiotensin receptors

Angiotensins are oligopeptides that exert their biological actions through the binding to specific angiotensin receptors (AT_1_, AT_2_, AT_3_, and AT_4_ receptors). It has been demonstrated that these receptors could be targeted for developing novel effective drugs for the treatment of hypertension, cardiovascular disorders, diabetic nephropathy, atherosclerosis (Goodfriend et al., 1996).

A series of symmetrically bis-substituted imidazole analogs has been designed based on docking studies, utilizing for the first time an extra hydrophobic binding cleft of the modeled AT_1_ receptor (Agelis et al., 2013). Four of the synthesized compounds showed high binding affinity to the AT_1_ receptor and high antagonistic activity (potency) similar or even superior to that of Losartan.

In an attempt to identify new AT_1_ receptor antagonists Pal and Paliwal (2012) developed a pharmacophore-based virtual screening protocol, which led to the identification of two active AT_1_ receptor antagonists with diverse structures (Pal and Paliwal, 2012).

### DRUG DESIGN AND DISCOVERY IN NEUROLOGICAL DISORDERS AND PAIN

#### Dopamine receptors

Dopamine exerts its function via five different receptors (D_1_, D_2_, D_3_, D_4_, and D_5_ receptors). This system plays a pivotal role in central nervous system and has been demonstrated to be involved in a series of neurological and psychiatric diseases such as Parkinson’s disease, schizophrenia, bipolar disorder, drug addiction, and Huntington’s disease (Pivonello et al., 2007; Beaulieu and Gainetdinov, 2011). The discovery of ligands able to modulate the dopaminergic system remains challenging and a lot of computational efforts were carried out for selecting potent and selective ligands.

In 2010, the crystal structure of D_3_ receptor was solved, which definitely confirmed the utility of homology models in GPCRs drug discovery (Chien et al., 2010). Indeed, Carlsson et al. (2011) docked over 3.3 million molecules against a homology model, and 26 of the highest ranking were tested for binding. Six had affinities ranging from 0.2 to 3.1 μM. Subsequently, the crystal structure was used and the docking screen repeated. Of the 25 compounds selected, five showed affinities ranging from 0.3 to 3.0 μM. One of the new ligands from the homology model screen was optimized reaching an affinity to 81 nM. The paper clearly demonstrated the feasibility of high throughput docking using modeled GPCRs.

The solved crystal structure of D_3_ receptor with a D_2_/D_3_ selective antagonist provides an opportunity to identify subtle structural differences between closely related GPCRs that can be exploited for novel drug design. In an elegant work Lane et al. (2013) performed virtual screening for orthosteric and putative allosteric ligands of D_3_ receptor using two optimized crystal-structure-based models. The authors employed in the computational protocol a receptor with an empty binding pocket (D_3_ receptor-APO), and a receptor in complex with dopamine (D_3_ receptor-Dopa). Potential hits retrieved by using the two models were submitted to biological evaluation and functional characterization. Pharmacological studies showed 14 novel ligands with a binding affinity better than 10 μM in the D_3_ receptor-APO candidate list (56% hit rate), and eight novel ligands in the D_3_ receptor-Dopa list (32% hit rate). Most ligands in the D_3_ receptor-APO model spanned both orthosteric and extended pockets and behaved as antagonists at D_3_ receptor. Among the identified ligands, one compound showed the highest potency of dopamine inhibition (IC_50_ = 7 nM). In contrast, compounds identified by the D_3_ receptor-Dopa model were predicted to bind an allosteric site at the extracellular extension of the pocket. Such compounds showed a variety of functional activity profiles. In fact, at least two compounds were non-competitive allosteric modulator of dopamine signaling in the extracellular signal-regulated kinase and β-arrestin recruitment assays. The high affinity and LE of the chemically diverse hits identified in this mentioned study evidently demonstrated the utility of structure-based screening in targeting allosteric sites of GPCRs (Lane et al., 2013).

Very recently, Vass et al. (2014) reported a prospective structure based virtual fragment screening on D_3_ and the H_4_ receptors. Representative receptor conformations for ensemble docking were obtained from molecular dynamics (MD) trajectories. Biological evaluation confirmed hit rates ranged from 16 to 32%. Hits had high LE values in the range of 0.31–0.74 and also acceptable lipophilic efficiency, demonstrating that the X-ray structure, the homology model, and structural ensembles were all found suitable for docking based virtual screening of fragments against these GPCRs.

#### Muscarinic receptors

The muscarinic acetylcholine receptors (M_1_–M_5_) are promising targets for the treatment of chronic obstructive pulmonary disease, urinary incontinence, and diabetes. Unfortunately, the lack of subtype specificity has remained a major obstacle to develop clinically useful muscarinic ligands. Very recently, Kruse et al. (2013) used the crystal structure of the M_2_ and M_3_ receptors as a template to identify, by means of structure-based docking, novel muscarinic ligands. Interestingly, one compound was a partial agonist at the M_3_ receptor without measurable M_2_ agonism that was able to stimulate insulin release from a mouse β-cell line (Kruse et al., 2013).

#### Cannabinoid (CB) receptors

The cannabinoid 1 receptor (CB1 receptor) and the cannabinoid 2 receptor (CB2 receptor) are members of the GPCR family (Matsuda et al., 1990). Agonists of both cannabinoid receptor subtypes produce strong antinociceptive effects in animal models of chronic, neuropathic, and inflammatory pain and are intensively investigated as potential new analgesic and antiinflammatory agents. CB1 antagonists are clinically established to be effective in treating obesity, obesity-related cardio-metabolic disorders, and substance abuse, but there are currently no marketed CB1 antagonists. The relevance of CB2-mediated therapeutics is well established in the treatment of pain, neurodegenerative, and gastrointestinal tract disorders (Di Marzo, 2008; Brogi et al., 2011; Pasquini et al., 2012).

Pandey et al. (2014) used homology model and high throughput docking to discover new chemical classes of CB1 antagonists that may serve as starting point for drug development. The authors developed and validated a homology model of CB1 based on a bovine rhodopsin template, which led to the discovery of seven compounds with an inhibitory potency >50% at 10 μM (Pandey et al., 2014). Wang et al. (2008) identified a novel class of azetidinones as CB1 antagonists by also using virtual screening methods. Meng et al. (2010) reported the identification of the benzhydrylpiperazine scaffold as a potential scaffold to develop novel CB1 receptor modulators using a privileged structure-based approach. The authors identified a highly potent and selective CB1 receptor inverse agonist that was able to reduce body weight in diet-induced obese Sprague–Dawley rats.

A recent work carried out by Renault et al. (2013) highlighted the importance related to crystallization of class-A GPCRs in a range of active states, identifying specific anchoring sites for CB2 agonists retrieved in an agonist-bound homology model of CB2 receptor. Docking-scoring enrichment tests of a high-throughput virtual screening of 140 compounds led to 13 hits within the μM affinity range. Interestingly, a relevant number of selected hits behaved as CB2 agonists, among them two novel unrelated full agonists were identified. Notably, the exclusive discovery of agonists illustrated the reliability of this agonist-bound state model in the discovery of GPCR ligands with desired behavior (Renault et al., 2013).

Recently, some of us described a three-dimensional quantitative structure–selectivity relationships (3D-QSSR) study for selectivity of a series of structurally diverse ligands characterized by a wide range of selectivity index values for cannabinoid CB1 and CB2 receptors (Brogi et al., 2011). 3D-QSSR explorations were expected to provide design information for the design of selective CB2 ligands. The computational model proved to be predictive, with *r*^2^ of 0.95 and *Q*^2^ of 0.63. In order to get prospective experimental validation, the selectivity of an external data set of 39 compounds reported in the literature was predicted by means of 3D-QSSR model (*r*^2^ = 0.56). Subsequently, a quinolone derivative predicted to be a selective CB2 ligand was synthesized and found to be an extremely selective CB2 ligand displaying high CB2 affinity (*Ki* = 4.9 nM), while being devoid of CB1 affinity (*Ki* > 10,000 nM). This finding confirmed that the ligand-based tool represent a valuable complementary approach to docking studies performed on homology models of GPCRs.

#### Opioid receptors

Opioids are key medications for the treatment of pain.The μ-opioid receptors (MORs), δ-opioid receptors (DORs), κ-opioid receptors (KORs), and nociceptin-opioid receptor (NOP) have been isolated and cloned. The receptors were found throughout the peripheral and central nervous system. Their important role in mediating pain, drug addiction, and depression has been established. Very recently crystal structures of all classes of opioid receptor have been solved (Granier et al., 2012; Manglik et al., 2012; Thompson et al., 2012; Wu et al., 2012; Fenalti et al., 2014). Below is reported one of the first computational efforts using the crystal structure of the KOR.

Negri et al. (2013) applied a structure-based computational protocol using the crystal structure of KOR receptor, discovering a selective novel KOR agonist, exhibiting analgesic effects without activating reward pathways. Remarkably, the novel derivatives have been identified as novel pharmacological tools to study the involvement of KOR in the etiology of drug addiction, depression, and pain (Negri et al., 2013).

## ALLOSTERIC MODULATORS AND BIVALENT LIGANDS

### ALLOSTERIC MODULATORS

The binding site of the endogenous agonist is qualified as orthosteric. In general, antagonists, and inverse agonists typically occupy also this site, which is usually buried at the core of the receptor or located at its extracellular N-terminal end. In addition, exist allosteric sites that bind synthetic drugs or endogenous mineral cations, such as sodium, calcium, zinc, and magnesium, which can also modulate the activity of the receptor (Christopoulos, 2002). More specifically, allosteric ligands may promote or reduce the binding of orthosteric ligands. Their effects on receptor activation could be in a positive, negative, or neutral manner. Allosteric modulators offer several advantages over classical approaches. Allosteric modulator can modulate affinity via conformational coupling between the orthosteric and allosteric binding sites or modulate efficacity by altering the functional response of the receptor to orthosteric ligand binding. These mechanisms can be dominant for a particular allosteric drug candidate and have significant value in the drug development process. Allosteric modulators can have a chemical structure unrelated to that of competitive agonist or antagonist drugs, offering a novel class of small molecule drug candidates.

The orthosteric binding sites within A class GPCR family are highly conserved due to the evolutionary pressure to retain amino acid sequences necessary for binding of the endogenous ligand. In contrast, allosteric modulator binding sites have much greater structural diversity than endogenous ligand binding sites, displaying a very high selectivity for a receptor subtype (Mohr et al., 2013).

**FIGURE 2:**
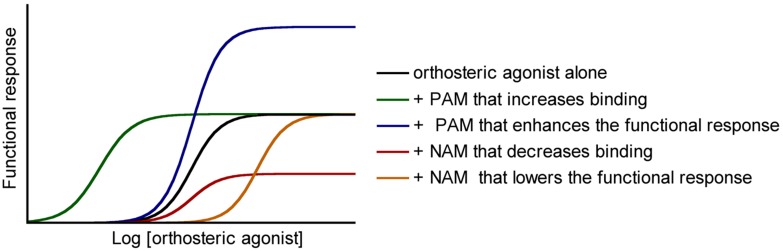
**Functional responses of allosteric modulators.** Positive and negative allosteric modulators (positive allosteric modulators and negative allosteric modulators) may modulate the affinity and/or the efficacy of orthosteric agonists.

Negative allosteric modulators (NAMs) bind at the allosteric binding site to inhibit the efficacy or affinity to the orthosteric binding site of the agonists while they have no intrinsic agonist efficacy. Two mechanisms can be invoked: the NAMs may stabilize an inactive conformation of the receptor that lowers the affinity of the agonists, or alternatively they raise the energy barrier necessary to activate the receptor activation, which diminishes the intensity of the output response (**Figure 2**, Burford et al., 2011).

On the very opposite, the binding of positive allosteric modulators (PAMs) to their allosteric binding site promotes the binding of the agonists at the orthosteric site or lower the barrier of energy involved in the shift to the active conformation of the receptor (Burford et al., 2011). The major drawback with this class of drugs is that they do not display any pharmacological effect in the absence of the endogenous (or exogenous) orthosteric agonist. Hence, a PAM in combination with an orthosteric agonist can increase the efficacy of the orthosteric compound. The PAM can allow a decrease in the dose administered, thereby improving the overall side-effect profile (**Figure 2**).

Silent allosteric modulators (SAMs) are neutral allosteric ligands. They have no effect on orthosteric agonist affinity or efficacy. However, SAMs can act as competitive antagonists at the same allosteric site, blocking PAM or NAM activity. SAMs can be effective tools to show that presumed PAM or NAM effects are receptor-mediated (Burford et al., 2013).

Interestingly, minor structural modifications are sufficient to transform a NAM into a PAM. Such a subtle effect have not been reported yet with class A GPCRs, even though it is likely that it will be found in a close future, due to the growing importance of this field of research. A striking example of this phenomenon concerns allosteric ligands of metabotropic glutamate receptors (mGluR5), class C family of the GPCRs, (**Figure 2**). While compound 1 is a partial NAM that only partially block mGluR5 signaling, introduction of a mere methyl can convert this compound to a full NAM or PAM (Williams et al., 2010).

**FIGURE 3:**
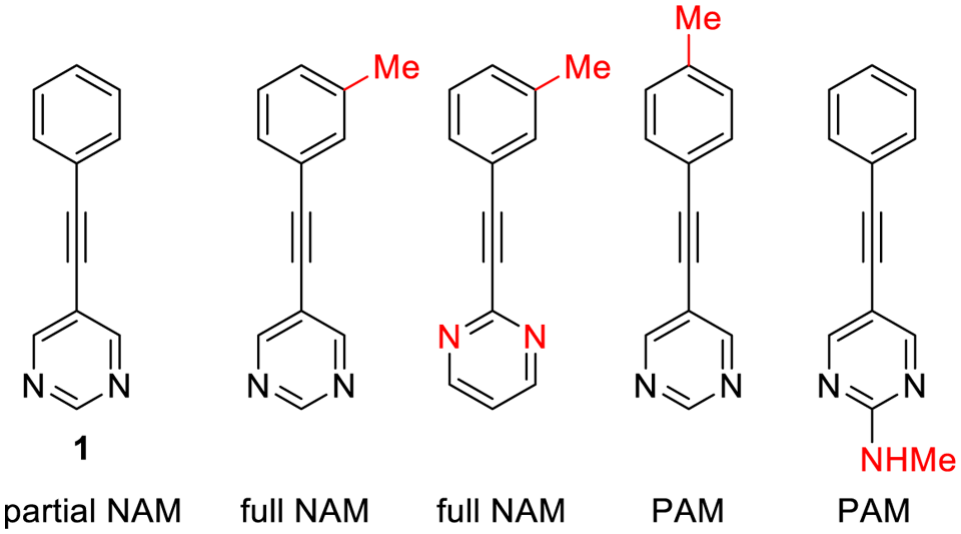
**Selected examples of mGluR5 allosteric ligands illustrating how a minimal structural variation can deeply affect the allosteric profile**.

Already two NAMs and one PAM have been approved for clinical use: Maraviroc (Celsentry), plerixafor (Mozobil), and Cinacalcet (Mimpara; **Figure 3**). Maraviroc is a high-affinity NAM of the CCR5 receptor that blocks the interaction of the HIV-glycoprotein 120 with this receptor (Fatkenheuer et al., 2005). It was approved in 2007 for the treatment of HIV in combination with antiretroviral agents. Plerixafor is a NAM of the chemokine receptor CXCR4. This medicine is used to promote the release stem cells into the bloodstream after autologous stem cell transplantation (Scholten et al., 2012).

Cinacalcet is a PAM of the calcium-sensing receptor (CaSR) of parathyroid hormone (PTH) producing cells. In a feedback mechanism, activation of CaSR by cinacalcet inhibits PTH release. This medicine was approved in 2004 for the treatment of secondary hyperparathyroidism in patients with chronic kidney disease on dialysis, and hypercalcaemia in patients with parathyroid cancer (Torres, 2006).

### MONOVALENT LIGANDS SPECIFIC FOR GPCR HETERODIMERS

**FIGURE 4:**
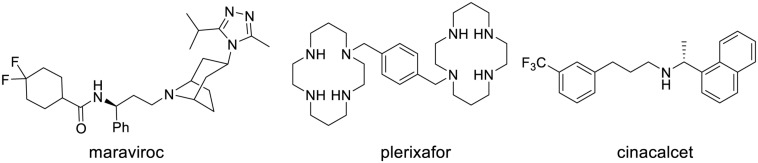
**Structure of approved allosteric modulators**.

**FIGURE 5:**
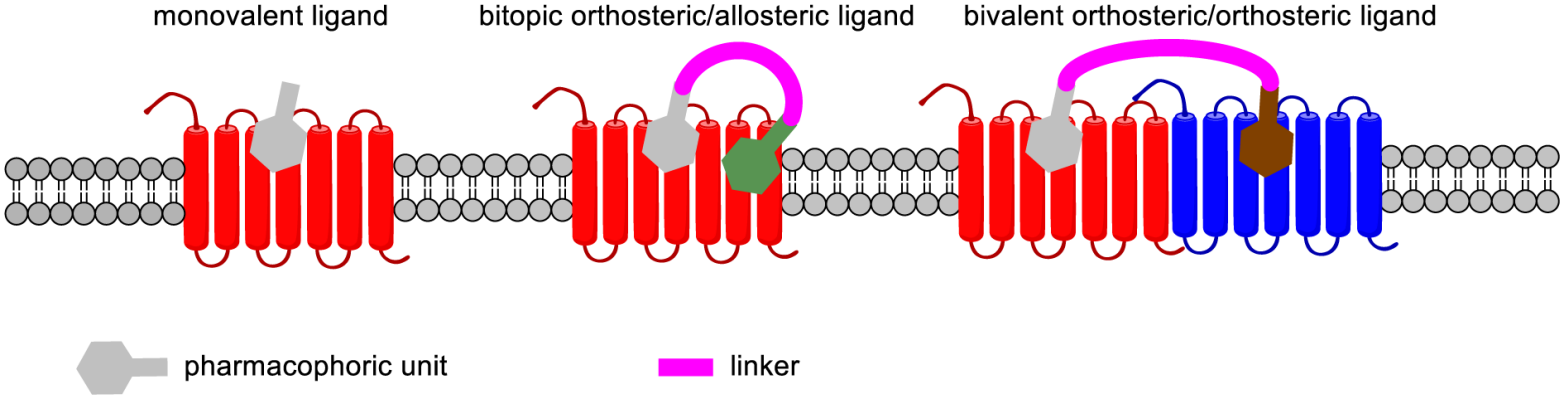
**Binding mode of monovalent, bitopic orthosteric/allosteric, and bivalent orthosteric/allosteric ligands of GPCRs**.

It is now well established that GPCRs may form homodimers, heterodimers, or oligomers. Even though their physiological function is not fully apprehended, these dimerizations and oligomerizations have major repercussions on ligand binding, activation of signaling pathways and cellular trafficking. Therefore, targeting specific GPCR dimers may provide drugs with enhanced potency, selectivity, and therapeutic index. Two types of such drugs that are specific for a specific GPCR dimer have been described (**Figure 4**). The first type concerns monovalent drugs, such as 6′-guanidinonaltrindole (6′GNTI), NNTA or SKF83959, that bind to only one receptor at a time. The second one concerns bivalent drugs that bind to two receptors at the same time (**Figures 4** and **5**).

Waldhoer et al. (2005) found that 6′-GNTI behaves as an extremely potent agonist in cells expressing both DORs and MORs and established that this drug selectively activates the δOR–κOR heterodimer (Waldhoer et al., 2005). *In vivo*, 6′-GNTI induces a potent analgesia when administered intrathecally. This δOR–κOR heterodimer was found to be expressed in a tissue selective fashion suggesting that such a drug may induce less side effects than classical OR agonists. Similarly, NNTA selectively activates the μOR–κOR heterodimer to induce a potent antinociceptive response devoid of physical dependence in mice (Yekkirala et al., 2011).

Another interesting example is provided by SKF83959 that selectively targets the D_1_–D_2_ dopaminergic heterodimer to increase intracellular calcium levels through activation of G_q/11_ (Rashid et al., 2007). Interestingly, this drug does not activate adenylyl cyclase, which is normally induced by the signaling of D_1_ or D_2_ receptors.

### BIVALENT LIGANDS

Different domains of GPCRs such as intracellular loops (ICL), extracellular loops (ECL), and transmembrane domains (TM) are known to participate in ligand recognition and receptor dimerization. Many GPCRs can form oligomers with conformational rearrangements of the receptors that impact their signaling (Percherancier et al., 2005).

**FIGURE 6:**
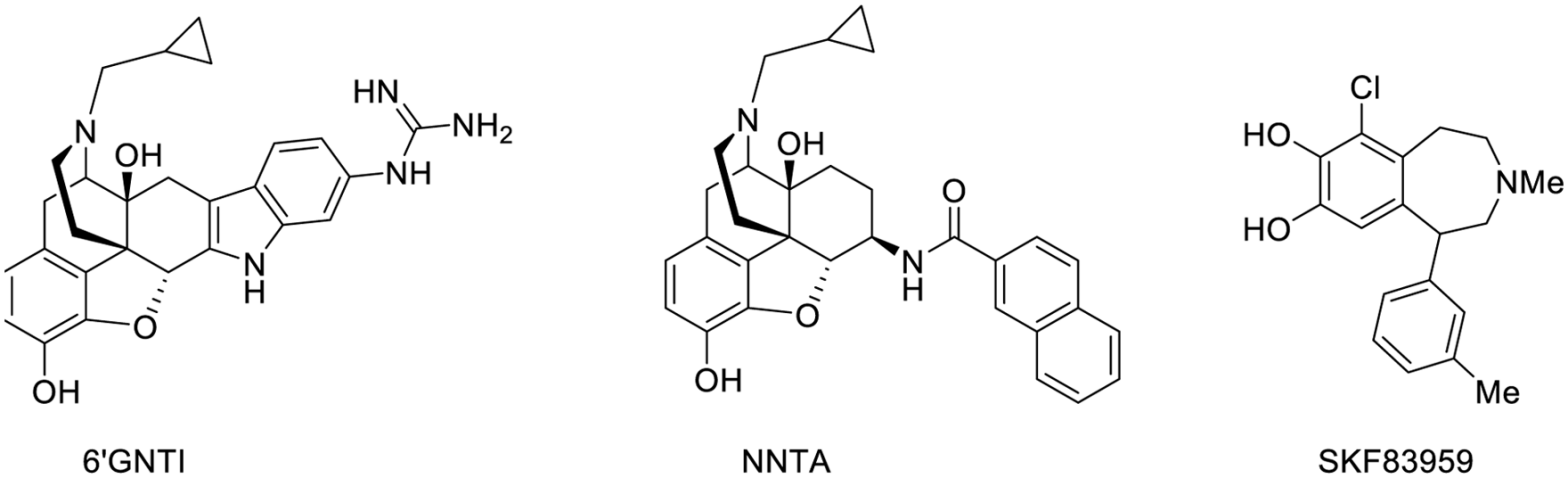
**Structure of monovalent drugs acting on GPCR dimers**.

Bivalent ligands are composed of two pharmacophoric units connected through a linker (while monovalent drugs encompass only one pharmacophoric unit). The pharmacophores may be identical (and in that case, the ligand is termed as homobivalent) or different in the case of heterobivalent ligands. These pharmacophoric units may either bind to the orthosteric site and an allosteric site within the same receptor or to two orthosteric binding sites located on two different receptors (**Figure 6**).

**FIGURE 7:**
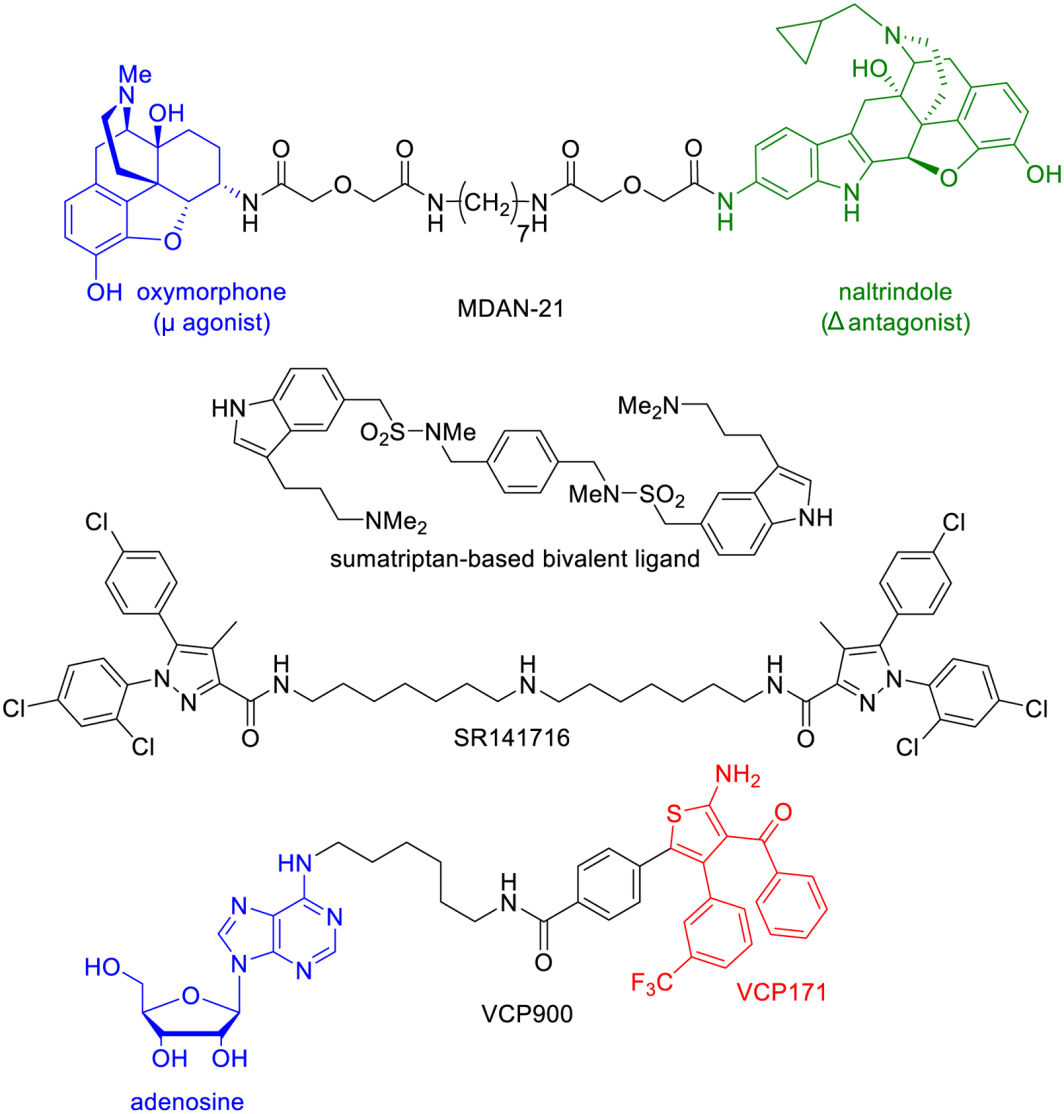
**Structure of bivalent ligands that display *in vivo* pharmacological activities**.

Often the large size and molecular weight of bivalent ligands severely reduce their bioavailability and hinder their use in *in vivo* studies. However, these limitations are not irretrievable and few bivalent ligands have shown interesting *in vivo* pharmacological activities, even though none of them entered a clinical trial (**Figure 7**).

Portoghese and colleagues have conjugated a μ agonist (oxymorphone) to a δ antagonist of opioids receptors (naltrindole) through a 21-atom linker (Daniels et al., 2005). The resulting compound, MDAN-21, was able to cross the blood brain barrier to induce antinociception. Impressively, this drug was 50 times more potent than morphine, and did not induce tolerance or physical dependence after chronic treatment.

In another example, Halazy and colleagues dimerized a 5-HT_1B_ agonist, sumatriptan, through a linker to obtain an orally active drug that induced a stronger hypothermia than sumatriptan itself (Perez et al., 1998). It is remarkable that such a drug could cross the blood–brain barrier in spite of its elevated molecular weight, polar surface area, and number of hydrogen-bond donors, suggesting that an active transport is probably involved.

SR141716 is another interesting bivalent drug that combines two units of a cannabinoid CB1 receptor antagonist/inverse agonist (Zhang et al., 2010). This compound was found to efficiently cross the blood–brain barrier to inhibit the antinociceptive effects of a cannabinoid agonist.

Very recently, Christopoulos and colleagues conjugated adenosine to VCP171, a PAM of the adenosine A1 receptor (A_1A_R; Valant et al., 2014). The obtained compound, called VCP746 binds to both the orthosteric and allosteric sites and behaves as a biased agonist (**Figure 7**). Importantly, it protects *in vitro* cardiomyoblasts and cardiomyocytes against simulated ischemia, but in contrast to classical A_1A_R agonists it does not perturb rat atrial heart rate *in vivo*.

## BIASED-SIGNALING SELECTIVITY

G protein-coupled receptors ligands are described by their efficacy (agonist, antagonist, partial agonist, or inverse agonist) and target (receptor type and subtype). Recently, great attention has been devoted to functional selectivity of GPCR ligands for the development of better therapeutic drugs with potentially fewer off-target and/or side effects. Ligand bias has been described based on their functional selectivity that preferentially signal through either G protein- or β-arrestin-mediated pathways.

Allosteric ligands can induce biased G protein signaling, thus representing interesting opportunities for drug discovery. Moreover, biasing β-arrestin-dependent signaling has also been shown to be potentially beneficial in heart diseases.

To delineate the contributions of G proteins and β-arrestins to GPCR function several approaches have been used including targeted genetic deletion of GRKs or β-arrestins, RNA silencing of G protein and β-arrestin, and small-molecule inhibitors of specific signal transduction pathways (DeWire et al., 2007). An important approach to investigate whether GPCR ligands are G protein-biased or β-arrestin-biased agonists is the use of β-arrestin knockout mice (Rominger et al., 2014). Indeed, the recapitulation of improved pharmacology in β-arrestin KO mice by a ligand demonstrates that this ligand is a G-protein biased ligand and may be particularly sensitive to the acute desensitization effects of β-arrestin. Inversely, minor pharmacological effects in β-arrestin KO mice indicate that β-arrestin is required for the specific intracellular signaling pathways of these β-arrestin-biased ligands.

**FIGURE 8:**
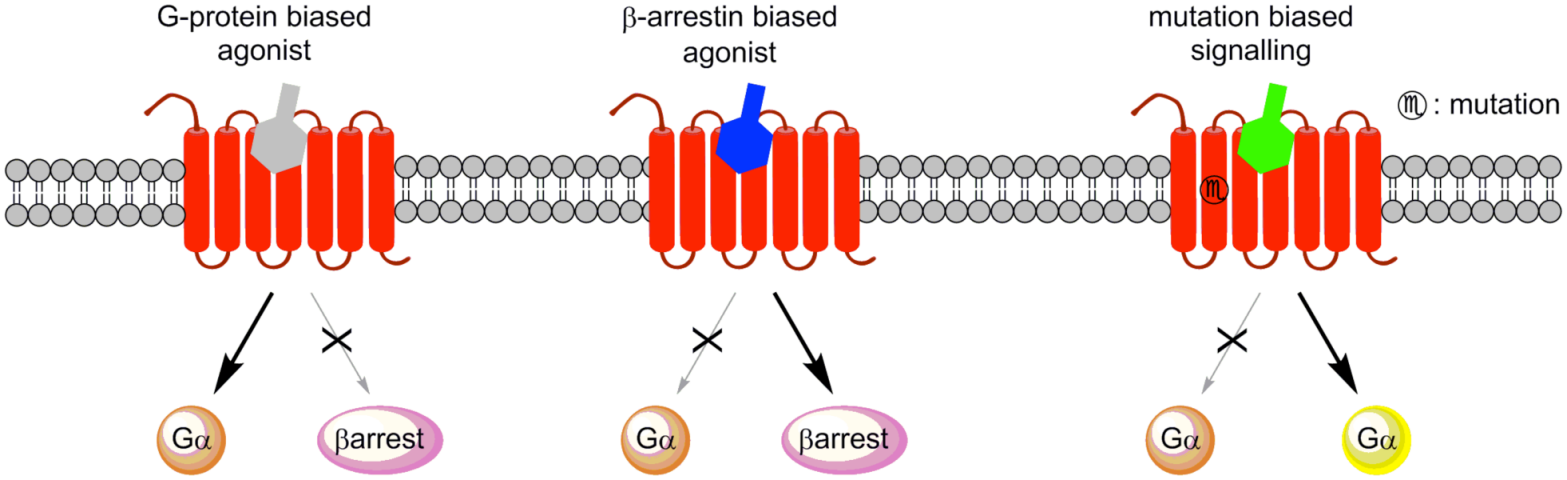
**Signaling of biased agonists.** G-protein biased agonists preferentially activate G-protein signaling. β-arrestin biased agonists activate β-arrestin signaling, and mutation mediated biased signaling may modify the G protein coupling.

Biased ligands that selectively activate β-arrestin signaling pathways over G_q_ G_i_ and G_s_-coupled GPCRs have already been reported (Violin and Lefkowitz, 2007; Gesty-Palmer et al., 2009). Biased signaling can also results from mutation of receptors (Leach et al., 2012; Sbai et al., 2014; **Figure 8**).

Another advanced approach is receptor activated solely by synthetic ligand’ (RASSLs). The chemical genetic approach involves the expression of a mutant form that can be activated by synthetic drugs but not by the endogenous ligands. For example a specifically mutated muscarinic receptor can be activated by clozapine-*N*-oxide (CNO), but not by acetylcholine (Armbruster et al., 2007). This approach has been utilized to determine GPCR signaling pathways important in β-islet function (Guettier et al., 2009), neuronal networks involved in neurological responses such as locomotion learning and memory (Garner et al., 2012), limbic seizures, and metabolism (Kong et al., 2012).

### G PROTEIN-BIASED MORPHINE μ-OPIOID RECEPTOR (MOR) LIGANDS

Both MOR and DOR are involved in analgesic effect of opioids. Thermal nociception is primarily modulated by MORs while mechanical nociception is primarily mediated by DOR (Scherrer et al., 2009), suggesting that these receptors are expressed in distinct circuits. Opioids cause postoperative nausea and vomiting, constipation, and sedation, giving significant patient discomfort, and can prolong hospital stay (Anastassopoulos et al., 2011). The respiratory suppression also limits opioid dosing, leaving many patients in pain during recuperation (Dahan, 2007). The classical μ opioid morphine increases efficacy and duration of analgesic response with reduced gastrointestinal dysfunction, and less respiratory suppression in β-arrestin-2 knockout mice compared to wild-type mice. This data clearly suggested that G protein-biased MOR agonists would be more efficacious with reduced adverse than current opioids.

The G protein-biased MOR agonist TRV130 has robust G protein signaling, with less β-arrestin recruitment and receptor internalization. TRV130 increases analgesia with reduced CNS depression and reduced gastrointestinal dysfunction compared with morphine. Thus TRV130 may provide a marked improvement over current opioids in postoperative care. It also holds great promise for chronic pain management, where constipation is a severe and often dose-limiting adverse event. TRV130 has been currently evaluated in human clinical trials for the treatment of acute severe pain (Chen et al., 2013). TRV130 displays broad dose margins between MOR-mediated pharmacology and intolerance in healthy volunteers (Soergel et al., 2014).

Similarly, a β-arrestin–MAPK pathway mediates stress and aversion-associated effects of kappa opioid receptor agonists, suggesting that biased kappa opioid ligands could provide analgesia without the dysphoric effects associated with classic kappa opioid agonists (Bruchas et al., 2010).

### β-ARRESTIN-BIASED DOPAMINE D_2_ LIGANDS

Dopamine plays a major role in reward-motivated behavior and motor control. The physiological actions of dopamine are mediated by five distinct but closely related GPCRs that are divided into two major groups: the D_1_ and D_2_ classes of dopamine receptors (Vallone et al., 2000). This classification is generally based on the original biochemical observations showing that dopamine is able to modulate adenylyl cyclase (AC) activity. Non-canonical modes of dopamine D_2_ receptor (D_2_R) signaling via β-arrestin is important for the therapeutic actions of both antipsychotic and antimanic agents. Aripiprazole, a FDA-approved atypical antipsychotic drug, was one of the first functionally selective D_2_R ligands identified (Urban et al., 2007; Mailman and Murthy, 2010). However, aripiprazole could behave as a full agonist, a partial agonist, or an antagonist at D_2_R depending on the cell type (Shapiro et al., 2003; Urban et al., 2007).

It was found that the antipsychotic action of an aripiprazole analog, UNC9975, was attenuated in the β-arrestin-2 knockout mice. UNC9975 also represents unprecedented β-arrestin–biased ligands for a G_i_-coupled GPCR. Significantly, UNC9975 is an antagonist of G_i_-regulated cAMP production and partial agonist for D_2_R/β-arrestin-2 interactions. Importantly, UNC9975 displayed potent antipsychotic-like activity without inducing motoric side effects *in vivo* (Masri et al., 2008). This β-arrestin–biased ligand shows a potent ability to suppress both *d*-amphetamine and phencyclidine-induced hyper locomotion in mice, indicating that it possesses antipsychotic activities *in vivo*. β-arrestin–biased ligands induce a lack of internalization. Thus, we can assume that drugs that induce internalization would ultimately foster tachyphylaxis and receptor down-regulation (Allen and Roth, 2011).

### MISSENSE MUTATION GPCR LEADING BIASED SIGNALING IN DISEASES

Many biased signaling are due to the ligand (Whalen et al., 2011), but few examples of biased signaling induced by a mutation of receptors have also been reported (Rajagopal et al., 2010).

A natural mutation leading to biased signaling has been identified in the thyroid stimulating hormone (TSH) receptor gene. The mutant TSH receptor still couples to G_s_ and activates cAMP but completely loses G_q_-mediated inositol phosphate production. This mutation on TSH receptor causes euthyroid hyperthyrotropinemia with increased radioiodine uptake (Grasberger et al., 2007).

Another example is the natural mutations in the human calcium sensing receptor that activate both G_q_-dependent production of inositol phosphate and the G_q_- and G_i/o_-dependent phosphorylation of ERK (Leach et al., 2012; Nygaard et al., 2013). It is generally assumed that biased signaling is an intrinsic property of a given ligand-GPCR complex, whereby a GPCR exists in several conformations, each of which is preferentially stabilized and activated by selective ligands (Nygaard et al., 2013). Likewise, the mutations leading to biased signaling are supposed to affect the equilibrium between the different receptor conformations.

The mutations in the GPCRs can lead to biased downstream signaling and may induce pathogenic and, in some cases, protective roles.

Prokineticins are anorexigenic and angiogenic hormones that couple to two GPCRs, PKR1, and PKR2 (Nebigil, 2009; Dormishian et al., 2013; Szatkowski et al., 2013). Mutations in the prokineticin receptor 2 (PKR2) have been found in 10% of patients with Kallmann syndrome that is characterized by hypogonadotropic hypogonadism. To date, 21 missense mutations of PKR2 have been identified in Kallmann syndrome patients. Some of these mutations are related with the G_q_-dependent signaling pathway (Sinisi et al., 2008; Abreu et al., 2012; Sbai et al., 2014). However, certain mutations on this receptor affect β-arrestin recruitment (R80C) or the G_q_ and G_i_ signaling pathways (R164Q) with normal G_s_ signaling. The G_q_-dependent signaling defect of the R164Q receptor makes this mutation most likely pathogenic. The mutation R268C affecting a residue in the third intracellular loop of the receptor selectively impairs G_i/o_-dependent signaling of the receptor and is considered non-pathogenic (Sbai et al., 2014). It remains unclear whether the β-arrestin-dependent signaling defect for the R80C mutation on PKR2 has a pathogenic effect with respect to Kallmann syndrome.

### BIASED LIGANDS IN DISEASES

Two GPCRs, the angiotensin II (AngII) type 1 receptor (AT_1_R) and the β-ARs are targets of widely used cardiovascular drugs. They are now potential therapeutic targets for biased ligands (DeWire and Violin, 2011).

The peptide hormone angiotensin II (AngII) is a vasopressor that regulates salt and fluid homeostasis, modulating vasoconstriction, and aldosterone secretion, as well as thirst and inflammation (Benigni et al., 2010). Angiotensin-converting enzyme inhibitors that lower AngII levels and angiotensin receptor blockers are widely used in treating hypertension and other cardiovascular diseases. The AT_1_R couples primarily to G_αq_ signaling, leading to phosphatidylinositol bisphosphate hydrolysis, generating diacylglycerol, mobilizing calcium, and activating signaling enzymes such as protein kinase C. AT_1_R is also involved in β-arrestin–dependent signals, activation of epidermal growth factor receptor transactivation, Src, and JAK/STAT (Saito and Berk, 2001; Wei et al., 2003; Oliveira et al., 2007). One body of evidence for distinct AT_1_R signaling came from receptor mutagenesis. AT_1_R effects can be divided into distinct G-protein–dependent and G-protein–independent signals *in vivo.* Reduction or elimination of β-arrestin-1 or β-arrestin-2 expression with siRNA *in vitro* or genetic deletion *in vivo* showed that cardioprotective effect of AT_1_R is mediated by β-arrestin-2 signaling. TRV120027, a selective and β-arrestin–biased AT_1_R ligand blocks AngII-dependent hypertension while increasing cardiomyocyte contractility, promoting cytoprotective, or antiapoptotic signals and preserving kidney function to provide a great benefit in acute heart failure (Monasky et al., 2013). TRV120027 is now in clinical trials for the treatment of acute heart failure (Soergel et al., 2014).

Endothelins play a key role in vascular homeostasis. ET_A_ and AT_1_ receptor antagonists both lower blood pressure in hypertensive patients. Accordingly, a dual ET_A_ and AT_1_ receptor antagonist may be more efficacious antihypertensive drug than current medicines.

Epinephrine binds to cardiac β_1_AR and stimulates inotropy through G-protein signals, resulting in increased heart rate, blood pressure, and metabolic stress, promoting cardiomyocyte apoptosis. Several studies demonstrated that β_1_AR G-protein and β-arrestin pathways normally strike a balance between apoptosis associated with prolonged inotropy and counteracting cardioprotection. When this balance is disrupted in the absence of β-arrestin signaling, apoptosis increases and cardiac function decreases. Activation of β-arrestin scaffolded calcium/calmodulin-dependent kinase II by the β_1_AR requires cAMP, thus the net effect of a β-arrestin–biased ligand is cardioprotective.

A biased ligand for β_1_AR, carvedilol activates the cardioprotective β-arrestin–mediated epidermal growth factor receptor transactivation-signaling pathway. Carvedilol has shown potentially superior clinical efficacy over other β-blockers in terms of cardiovascular events after myocardial infarction (Kopecky, 2006) and perhaps mortality (Poole-Wilson et al., 2003). The contributions of function of GRK/β-arrestin to the clinical efficacy of carvedilol remain unclear.

Collectively, substantial data suggest that biased ligands will have distinct and perhaps more beneficial effects than unbiased agonists. Biased signaling is proposed to be useful in several diseases, including heart failure (β-ARs), hypertension (α-ARs), neuropsychiatric and/or neurodegenerative disorders (histamine H_1_ receptors), schizophrenia, Parkinson’s disease (dopamine receptors), psychosis and depression (serotonin receptors), hypothyroidism (TSH receptor), hyperlipidemia (nicotinic acid receptor), diabetes (GLP1). However, it is possible that biased signaling could be associated with undesirable side effects and even contribute to disease. For example, the bacterium *Neisseria meningitidis* interacts in a biased and allosteric manner with the β_2_AR to initiate signaling cascades that facilitate meningeal colonization (Brissac et al., 2012).

## CONCLUSION

A substantial increase in our understanding of GPCR pharmacology has provided an array of ligands that target both orthosteric and allosteric sites of GPCRs as well as ligands that have properties of bias stimuli. The recent identification of a PAM and NAM binding site, together with the synthesis of *in vivo* effective ligands, represents a novel, and likely more favorable, option for pharmacological manipulations of the GPCRs. Biased ligands offer safer, better-tolerated, and more efficacious drugs. However, in some cases a path to successful drug development for targets that have been abandoned because of on-target adverse pharmacology in the clinical proof-of-concept studies due to additional receptor signaling and regulatory mechanisms rather than β-arrestin pathway.

The complexity of GPCR signaling requires a synergistic role for experimental and computational methods in producing novel therapeutics with maximal clinical efficacy and lowest toxicity. Combining computational methods with sophisticated transgenic and chemical genetic animal models, the next generation of GPCR ligands will unquestionably employ rational design principles to deliver GPCR ligands with minimal side-effects.

## Conflict of Interest Statement

The authors declare that the research was conducted in the absence of any commercial or financial relationships that could be construed as a potential conflict of interest.
